# Effects of Prices on Youth Cigarette Smoking and Tobacco Use Initiation in Ghana and Nigeria

**DOI:** 10.3390/ijerph16173114

**Published:** 2019-08-27

**Authors:** Samuel Asare, Michal Stoklosa, Jeffrey Drope, Aidan Larsen

**Affiliations:** 1 Department of Economics, Andrew Young School of Policy Studies, Georgia State University, Atlanta, GA 30303, USA; 2 Economic and Health Policy Research, American Cancer Society, Atlanta, GA 30303, USA; 3 Department of Health Policy and Management, Rollins School of Public Health, Emory University, Atlanta, GA 30322, USA

**Keywords:** cigarette smoking, tobacco use initiation, cigarette prices, price elasticity, cigarette taxes

## Abstract

*Background*: Population growth in the African region is set to outpace the rate of decline in smoking prevalence, leading to a projected increase in the total number of smokers. As most tobacco users initiate during their adolescent years, tobacco prevention strategies targeting youth will be particularly important. *Methods*: This study estimated the impact of cigarette prices on youth cigarette smoking and tobacco use initiation in Ghana and Nigeria using the Global Youth Tobacco Survey data. First, we used cross-section data and logit models to estimate the effects of prices on youth cigarette smoking. Second, we created pseudo longitudinal data and used continuous-time hazard models to evaluate the impact of cigarette prices on tobacco use initiation. *Results*: We found that higher cigarette prices decreased both 30-day cigarette smoking and tobacco use onset significantly in both Ghana and Nigeria. Additionally, the price elasticity of cigarette smoking and tobacco use initiation ranged from −0.44 to −1.13, and −1.04 to −3.66, respectively. *Conclusions*: As one of the first studies on youth tobacco consumption in Sub-Saharan Africa, this study strongly suggests that policies that increase real cigarette prices can lower both cigarette smoking and tobacco use initiation among youth in Ghana and Nigeria.

## 1. Introduction

Although tobacco smoking has declined worldwide, much of this decline has occurred in high-income countries (HIC), and the majority of current smokers now live in low- and middle-income countries (LMIC) [1]. As a result, 80% of tobacco-related deaths are predicted to occur in LMIC by 2030 [2]. However, in the World Health Organization’s Africa region (WHO-AFRO), population growth is set to outpace the rate of decline in smoking prevalence, leading to a projected increase in the total number of smokers in the region from 66 million in 2015 to 84 million in 2025 [3], the second-largest increase in the number of smokers among the WHO regions, after the Eastern Mediterranean (EMRO) region. 

Ghana and Nigeria are no exceptions to this pattern, with relatively low current smoking prevalence (7.0% and 10.6% for males and 0.2% and 0.6% for females, respectively, in 2016) [3] but with the number of smokers predicted to more than double from 2010 to 2025 (to 1.7 million and 16.7 million, respectively, in 2025) [4]. With a population of over 190 million people, Nigeria is currently the world’s seventh most populous country but is predicted to become the third most populous by 2050 [5]. Similarly, the population of Ghana is predicted to grow from the current 30 million to 52 million in 2050 [5]. Improving tobacco control policies in Ghana and Nigeria can stave off an emerging tobacco epidemic on a large scale [6]. Moreover, recent surveys have found smoking prevalence in both countries to be higher among young adults (25–29 years old) than among older cohorts (30–39 years old) [7,8]. As of 2017, an estimated 44% of Nigerians and 39% of Ghanaians are aged 14 or younger [6], suggesting that tobacco prevention strategies targeting youth will be particularly important.

Like much of Africa [9], tobacco control policies in Nigeria and Ghana have lagged behind the rest of the world [1]. Tobacco taxes in these two countries are at relatively low levels. As of 2016, both Nigeria and Ghana ranked in the bottom half of all African countries for overall tax on cigarettes [1]. Before 2018, Nigeria had a 20% ad valorem tobacco excise tax rate. In 2018, the country introduced a new specific excise tax of ₦ 1 per stick (₦ 1 = USD 0.028), which will grow to ₦ 2.90 in 2020. Even with this increase, the excise tax burden of the retail price will be only 17% [10], which is well below the WHO-recommended 70% minimum excise tax burden and the current global average of 45%. Ghana has similarly low excise tax rates, with excise tax comprising an estimated 13.0% of the retail price and no recent efforts to increase the tax [1]. The relatively low prices of cigarettes in both countries suggests large room for tax increases, which could be used to counter the very high likelihood of significant increases in the number of tobacco users in both countries.

Studies from multiple Sub-Saharan African countries [11], as well as specific-country studies, including from South Africa [12], Tanzania [13], Uganda [14], and Zambia [15], have confirmed that higher cigarette prices reduced smoking prevalence and intensity of use. Previous international research has found that youth are even more price-responsive than adults [16], making tobacco taxation particularly effective in countries with high population growth, where measures aimed at youth smoking are especially needed. However, strong evidence on the impact of taxes on smoking onset is still scarce [17]. There has been only one peer-reviewed study examining the relationship between prices and smoking onset in Sub-Saharan Africa [18]. That study used the 2008–2012 data from the National Income Dynamics Study in South Africa and found that while the initiation peaked at ages from 17 to 18 years, cigarette prices significantly reduced regular smoking initiation among males, but not among females. This current study adds to the literature by estimating the effect of cigarette prices on youth smoking and tobacco use initiation in Nigeria and Ghana.

## 2. Materials and Methods

### 2.1. Materials

We used several sources of data for this study. The Global Youth Tobacco Survey (GYTS) was our main data source [19]. The WHO has designed GYTS as a global standard for monitoring youth tobacco use and tracking key tobacco control indicators to guide the implementation and evaluation of tobacco prevention and control programs [20]. The GYTS is a cross-sectional survey of students in junior secondary schools (grade 7–9). (Some countries such as Nigeria, etc., include first-year students of senior secondary schools. Students included in the surveys were aged 11–18) Ghana has had four (2000, 2006, 2009, and 2017) waves of GYTS, but the data from the 2017 survey were still not available at the time of manuscript submission. Each wave surveyed students nationwide. In each survey from Ghana, a two-stage cluster sample design was used to produce representative data for the country [21]. In contrast, Nigeria has had only two (2000 and 2008) waves, which provided regional (state) data only: the 2000 data were for Cross River State and Abuja in the 2008 data. The survey response rates were high, with 83% in the 2000 wave [22], 86% in the 2006 wave [23], and 84% in 2009 data [21] in Ghana and 85% in 2000 data [24] and 89% in the 2008 wave [25] in Nigeria. 

#### 2.1.1. Dependent Variables

The dependent variables were current cigarette smoking and tobacco use initiation drawn from the GYTS data [19]. The respondents were asked to provide answers to their 30-days cigarette consumption patterns (“During the past 30 days (one month), on how many days did you smoke, how many cigarettes did you smoke?”). Current cigarette smoking was a dichotomous variable for smoking in the 30 days before the survey date.

To obtain the longitudinal data format needed for the duration analysis in the study of tobacco use initiation, we retroactively inferred the year of cigarette initiation using the GYTS question on the age of smoking initiation (“How old were you when you first tried a cigarette?”). This allowed us to obtain a pseudo-longitudinal dataset. The dataset was created with the assumption that individuals were exposed to the risk of initiation, starting from the age of eight, which is in line with a similar, previous study from Argentina [26]. In each period, the at-risk students received a value of 0 if they did not initiate. A given student was dropped out of the sample after initiation occurred. Our dataset spanned from 1995 to 2009. 

#### 2.1.2. Independent Variables

Our primary explanatory variable was cigarette price. We used two sources for cigarette price. First, we used the self-reported price of a pack of 20 cigarettes from the GYTS [19] (“How much do you usually pay for a pack of 20 cigarettes?”). These prices were calculated for survey years before 2007 only since GYTS discontinued collecting data on self-reported price after 2007. Therefore, we only had price data for the 2000 and 2006 waves in Ghana and the 2000 wave in Nigeria. One obstacle to using this price measure in product demand estimations was that these prices may have been endogenous in the models from the simultaneity of price and consumption. To address potential endogeneity, we averaged the prices by geographical regions, in each case excluding the self-reported price of that particular student. Specifically, for student *i,* in the primary sampling unit (*PSU*) *p* of size *N_p_*, surveyed in time *t*, the individual demand price was

(1) $$Price_{ipt}=\;\frac{1}{N_{p}-1}\sum_{j\neq i}^{N_{p}-1}Price_{jpt}$$

The second measure of price was the national-level price of cigarettes in the years from 1995 to 2009, constructed from different sources for each country. In the case of Nigeria, we used the price data collected and organized by The Economist Intelligence Unit [27] and adjusted it by the consumer price index from Nigeria’s National Bureau of Statistics [28]. In the case of Ghana, cigarette price data were not available from the Economic Intelligence Unit. We overcame this data limitation by constructing a price measure using WHO’s published prices of a pack of 20 cigarettes in the year 2010 and adjusting it by the consumer price indexes (CPI) for tobacco products taken from the Ghana Statistical Service [29] and International Monetary Fund Country Report on Ghana [30] (In Ghana, the prices are calculated as follows: $Price_{t-1}=\;\frac{1+\Pi_{Tob,t-1}}{1+\Pi_{All,t-1}}\times Price_{t},$ where $Price_{2010}=GHC2.00$, and $\Pi_{Tob}$ and $\Pi_{All}$ represented inflation for tobacco products, and consumer goods and services respectively). The source of variation in these prices come from changes in the real price of cigarettes over time. 

We included the following variables in our regressions as covariates: sex, age, grade (Grade variable included in addition to the age variable, as children in Ghana and Nigeria start their education at different ages), parents’ smoking status (at least one parent smokes vs. parents do not smoke), monthly pocket money (categories were provided by GYTS. We determined these categories as follows: lowest if student was not provided any pocket money, low if pocket money was less than ¢1000 and in the 2000 survey in Ghana, less than ¢11,000 and in the 2006 survey in Ghana, or less than ₦ 100 and in the 2000 survey in Nigeria, high if pocket money was ¢1000–¢2,900 and in the 2000 survey in Ghana, ¢11,000–¢29,000 and in the 2006 survey in Ghana, or ₦ 100–₦ 1000 and in the 2000 survey in Nigeria, and highest if pocket money was greater than ¢2900 and in the 2000 survey in Ghana, greater than ¢ 39,000 and in the 2006 survey in Ghana or greater than ₦ 1000 and in the 2000 survey in Nigeria), exposure to anti-smoking media messages and campaigns, classroom discussion about the harmfulness of tobacco use, having ever been offered a free cigarette by a cigarette representative, and last 30-day exposure to cigarette advertisements and promotions. In the pseudo-longitudinal dataset, we assumed that the values of the covariates did not change throughout the study period, except for the income, age, and grade variables. Therefore, instead of the pocket money variable, in the models using pseudo-longitudinal data, the income was approximated by per capita gross domestic product (GDP) [31]. A consequence of the time-invariant covariates was that the effects of the parameters and the covariates on the hazard rate were constant throughout the study period [32]. Ideally, we expected the hazard rate to change with respect to changes in these covariates. 

For the two-country analysis, we adjusted these prices and per capita GDP to constant Purchasing Power Parity (PPP)-adjusted dollars using the adjustment factor from the World Bank [33].

### 2.2. Methods

To estimate the effect of cigarette prices on current cigarette smoking, we used logit models, regressing current cigarette smoking on prices and other covariates. We estimated three models: one for Ghana, one for Nigeria, and one for the pooled data from both countries (i.e., two-country analysis). In each model, we clustered standard errors at the PSU level. We limited our sample to include data from 2000 and 2006 for Ghana and only 2000 for Nigeria, the years for which self-reported price was available. The models used the sample weights that were available with the data.

A survival model (continuous-time hazard model) in the complementary log-log (cloglog) functional form was used to estimate the effect of cigarette prices on tobacco use initiation. Cloglog has an asymmetric response curve and, therefore, was more appropriate to analyze our samples with a larger asymmetry toward nonsmokers than the standard probit and logit models [26]. Individuals who were already smokers before the study period were left-censored. On the other hand, individuals who did not initiate tobacco use throughout the study period were right-censored. Three models for Ghana, Nigeria, and the two-country analysis were, again, estimated using the weights provided with the data. Because the cloglog model is non-linear, we reported the marginal effects and their standard errors for interpretation.

One assumption of survival models was that all the individuals eventually failed—initiated to use tobacco products. Given that a large proportion of our sample never initiated throughout the study period, we used continuous-time split-population models as robustness checks. Unlike cloglog models, split population models could account for many failures in the sample. Unfortunately, estimating split-population survival models in Stata/MP 15 (StataCorp. 2017. *Stata Statistical Software: Release 15*. College Station, TX: StataCorp LLC, USA) did not have the option to include sampling weights or compute derivatives (marginal effects). To make meaningful comparisons, we estimated the cloglog model without sampling weights and reported their estimates without derivatives to compare with the estimates from the split-population models. 

## 3. Results

### 3.1. Effects of Cigarette Prices on Current Cigarette Smoking 

The total number of students in the samples were 9432 and 1555 for Ghana and Nigeria, respectively. In Ghana, about 90% of the students were surveyed in 2006. Table 1 summarizes all the variables for each country. On average, about 7.5% of students in Ghana were current cigarette smokers, compared to 8.7% of student in Nigeria. For both countries, the prevalence of current smokers in our combined sample was 7.7%. The average age of initiation in Ghana among users was 10, and in Nigeria, it was 12. The average inflation-adjusted price of a 20-pack of cigarette reported in Local Currency Unit (LCU) in Ghana was about GH¢ 0.87 (in 2006 prices) and ₦ 36.5 in Nigeria (in 2000 prices). These prices are equivalent to 80 cents and 35 cents in 2006 and 2000 U.S.$, respectively (We used the inter-bank exchange rate between U.S. and Ghana [34] and Nigeria [35]). Using PPP-adjusted factors, we converted the prices to 2006 international dollars (I$). Both prices were similar on average, and the average price in both countries was about 2.49 PPP-adjusted, international dollars (I$).

The first panel of Table 2 reports the marginal effects of the logit models, which regressed the last 30-day cigarette smoking on prices and other covariates. The estimates from all the models suggest that cigarette prices affected youth cigarette smoking in Ghana, Nigeria, and both combined and that this relationship is statistically significant. In Ghana, a 10 pesewas (≈9 U.S. cents) increase in the inflation-adjusted price of a pack of cigarettes reduced current cigarette smoking by 0.8 percentage points (approximately 10.7% reduction). Similarly, in Nigeria, a ₦ 1 (≈1 U.S. cent) increase in the inflation-adjusted price of a cigarettes pack reduced current cigarette smoking by 0.09 percentage points (approximately 1.0% reduction). In the two-country analysis, our estimates suggest that a I$1 increase in PPP-adjusted price of a pack of cigarettes reduced current cigarette smoking by 2.2 percentage points (approximately 28.0% reduction). A I$1 increase was an approximately 39% increase in the PPP-adjusted price.

To supplement our analysis, we computed the point price elasticity of demand for the last 30-day cigarette smoking. Panel B in Table 2 reports our estimates. Our results suggest that cigarette price elasticity was highly elastic (≈−1.13) in Ghana, fairly elastic in Nigeria (≈−0.44) and close to unitary elastic for both countries combined. We interpreted the price elasticities as follows: a 1% increase in cigarette prices decreased cigarette consumption among youth by 1.13% and 0.44% in Ghana and Nigeria, respectively. In the two-country analysis, a 1% increase in cigarette prices decreased cigarette smoking by 0.9%.

### 3.2. Effects of Cigarette Prices on Tobacco Use Initiation

**Figure 1:**
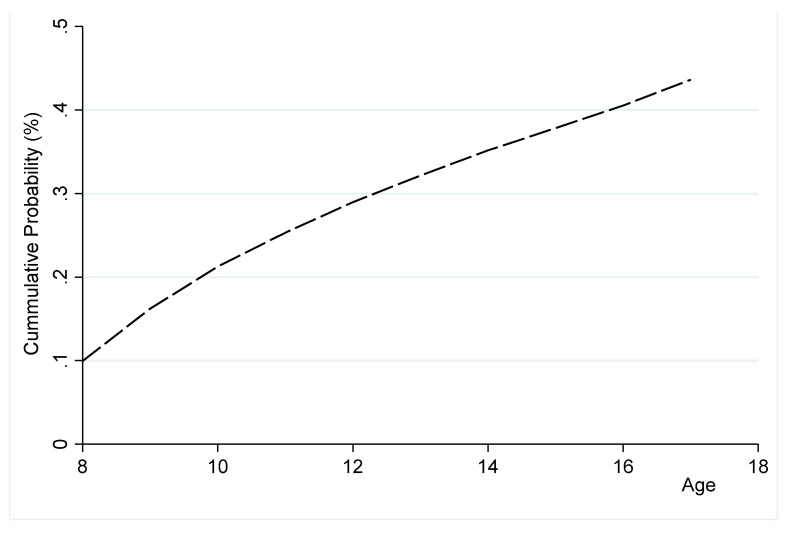
Cumulative risk of initiation into the use of tobacco products among youth (ages 8–17) in Ghana.

**Figure 2:**
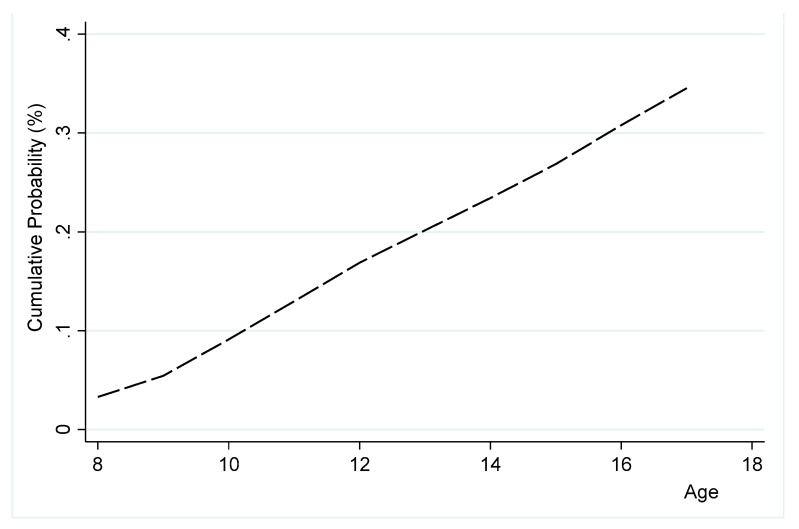
Cumulative risk of initiation into the use of tobacco products among youth (ages 8–17) in Nigeria.

We used all the survey samples of GYTS data from Ghana and Nigeria with a sample size of 18,231 and 2678, respectively. In the panel data that we generated, the number of observations in Ghana and Nigeria was 107,880 and 15,669, respectively. In Table 3, we summarized the models’ variables. The table shows that approximately 5% and 3.3% of students in our sample initiated in Ghana and Nigeria, respectively. Figure 1 and Figure 2 show the trends in the cumulative risks of initiation into using tobacco products in Ghana and Nigeria, respectively. They show that the accumulative risk of initiation in both countries increased sharply over the age profile. Students in Ghana accumulated more than 40% risk of initiation before age 16. Students in Nigeria faced a lower accumulated risk at age 16 compared to Ghana.

The average inflation-adjusted tobacco price in Ghana was GH¢ 1.76, and that of Nigeria was approximately ₦ 277 in 2008 prices. These prices are equivalent to U.S.$ 1.47 and U.S.$ 2.33 in 2008-dollar values. The average PPP-adjusted price for a 20-pack of cigarette in both countries was I$ 4.2. In Table 3, we also reported the statistics for all the covariates used in the models. 

The first panel of Table 4 presents the results of the continuous-time complementary log-log (cloglog) survival models. Our estimates from the data from Ghana suggest that a 10-pesewas (≈8 U.S. cents) increase in the price of a pack of cigarette reduced the risk of initiation by 0.91 percentage points (approximately an 18.4% reduction). In Nigeria, a ₦ 1 (≈0.1 U.S. cents) increase in the price of a pack of cigarettes reduced the risk of initiation by 0.015 percentage points (approximately a 0.46% reduction). Both estimates are statistically significant at 1%. Given that the average price of a pack of cigarette in Ghana and Nigeria were GH¢ 1.76 and ₦ 277 respectively, then the 10-pesewas and ₦ 1 increase represented a 5.7% and 0.35% increase, respectively. In the two-country analysis, our estimates suggest that a I$ 0.10 increase in a price of a pack of cigarettes reduced the risk of initiation by 3.6 percentage points (approximately 7.6% reduction) and was statistically significant at 1%.

We completed our results with some computations of price elasticity of demand for initiation. The second panel of Table 4 presents results on the price elasticities. Our results show that the price elasticities of demand for initiation in Ghana and Nigeria were highly elastic. In each country, as well as in the combined countries, the elasticities were greater than one in absolute terms. This suggests that every one percent increase in cigarette prices led to more than a one percent decrease in the risk of initiation.

### 3.3. Robustness Checks

We estimated the survival models using split-population survival hazard functions and reported the estimates without their derivatives. In Table 5, we reported the estimates from the cloglog and split-population survival models side by side for comparison. We did not find any significant differences between the two estimates for the independent variables. Although the estimates were consistently smaller in absolute terms when we used the split-population survival functions, we do not think the differences are an issue to address since the differences were small in magnitude.

We reported our results from the country-specific dataset in terms of the international dollars as the main independent variable in Table 6. With cigarette smoking as an outcome, we found that a I$ 1 increase in the price of a pack of cigarette reduced smoking by 2.2 and 1.5 percentage points (approximately 29.3% and 17.1%) in Ghana and Nigeria, respectively, and are statistically significant at 1%. We also found from the survival models with tobacco use initiation as a dependent variable that a I$ 1 increase in the price of a pack of cigarette reduced initiation by 4.7 and 0.7 percentage points (approximately 94.4% and 14.8%) in Ghana and Nigeria, respectively.

## 4. Discussion

This article is one of the first studies on the impact of cigarette prices on youth smoking and smoking initiation in Sub-Saharan Africa. We found that higher cigarette prices decreased both 30-day cigarette smoking and cigarette smoking onset in both Nigeria and Ghana. Our findings strongly suggest that further cigarette excise tax increases large enough to increase cigarette prices in Ghana and Nigeria will lower smoking rates and decrease smoking initiation among youth. Therefore, government policies aiming at reducing tobacco use among the youth must incorporate further cigarette tax increases. The positive impact of tax increases on public health multiplies when a portion of government revenue from tobacco is dedicated to health programs. Additionally, countries need to implement measures to prevent illicit trade in tobacco products. The tools to combat cigarette smuggling globally are provided by the Protocol to Eliminate Illicit Trade in Tobacco Products. While Nigeria ratified the Protocol in 2019, Ghana has yet to ratify the treaty.

The estimated price elasticity of smoking participation ranged from −0.44 to −1.13. These findings are similar to estimates from other LMIC [36] and are line with previous studies, which suggest high sensitivity to tobacco product prices among youth [16]. Our estimates of price elasticity for tobacco use initiation (−1.04 and −3.66 for Nigeria and Ghana, respectively) were higher than estimates from similar studies from LIMCs [18,37]. This is likely due to very low initiation rates in our sample: 5.0% and 3.3% in Ghana and Nigeria, respectively. With these low initiation levels, even a slight absolute change in the initiation level yields large relative changes. Our study also suggests that higher pocket money was associated with a higher likelihood of smoking among youth, an effect similar to studies on adult cigarette smokers, which generally find positive income elasticities [16].

There are other significant findings in this study. First, we found that students who were exposed to cigarette advertisements and who were offered free cigarettes were more likely to smoke. This suggests a need for governments to address these tobacco industry tactics. Second, students who knew about tobacco harms and had class discussions on the dangers of tobacco use were less likely to smoke, which attests to the effectiveness of student education. Third, students with a smoking parent were more likely to smoke themselves. This suggests that smoking habits transmit intergenerationally and that those education efforts should be especially focused on students who are most prone to smoke. Our finding that being exposed to anti-smoking media messages was associated with a lower likelihood of smoking is consistent with our expectation and that of previous studies [30]. It suggests that anti-smoking media messages (e.g., having a TV in the household) can be a channel for reducing cigarette smoking.

Our study had several limitations. The self-reported price used in the models of smoking likelihood was subjected to recall bias and can be endogenous. We attempted to mitigate both issues by averaging the price by PSU. Second, the reported age of initiation can be subject to recall bias, although this problem is likely not large, due to the young age of respondents. Third, the relative unbalanced samples from the countries bias the results from the pooled sample analysis towards the country with the large dataset (Ghana). In addition, the two Nigerian states for which the data was available are not necessarily representative of the country. The economy of the Cross River State is, to a larger extent, based on the industry sector, compared to the rest of the country, while services dominate in the Federal Capital Territory (Abuja). Per capita, GDP is also a bit higher in the Cross River State and much higher in Abuja than in the rest of Nigeria [38]. Finally, the cloglog model used to estimate the risk of initiation assumes that every respondent will smoke eventually. However, the split population model, which lifts this assumption, produced similar results. Thus, our models produced estimates of the price effects that were of expected magnitude and sign.

## 5. Conclusions

Consistent with evidence from high-income countries, this study affirms that higher cigarette prices in Ghana and Nigeria will lead to lower smoking rates and lower smoking initiation rates among youth in those countries. Both countries committed to implementing high excise taxes on tobacco products to reduce tobacco consumption when they became Parties to the WHO Framework Convention on Tobacco Control in 2004–2005, but the countries have been lagging on fulfilling this promise. Higher tobacco taxes leading to higher tobacco product prices will not only improve public health, particularly by protecting the health of the youth but also result in higher government revenue that might be further re-invested toward growth-enhancing sectors such as education or healthcare, further amplifying the benefits of tobacco taxes.

## Figures and Tables

**Table 1:** Characteristics of youth (ages 11–18) for a 30-day cigarette smoking (standard deviations in parentheses).

Variables	Ghana Only Using GYTS 2000 And 2006 Dataset (*N* = 9432)	Nigeria Only Using GYTS 2000 Dataset (*N* = 1555)	Both Countries Using GYTS 2000 And 2006 for Ghana, And GYTS 2000 for Nigeria (*N* = 10,987)
	Dataset (1)	Dataset (2)	Dataset (1) + Dataset (2)
Cigarette Smoking	7.52%	8.68%	7.68%
	(0.264)	(0.282)	(0.266)
Inflation—Adjusted Price (in LCU)	0.873	36.52	–
	(0.0807)	(12.68)	
PPP—Adjusted Price (in I$)	2.548	2.138	2.490
	(0.262)	(0.743)	(0.397)
At least One Parent Smokes	12.3%	12.2%	12.3%
	(0.329)	(0.328)	(0.329)
Gender (Male = 1)	52.1%	51.3%	52.0%
	(0.500)	(0.500)	(0.500)
*Age*			
Age 11–12	19.1%	14.0%	18.4%
	(0.393)	(0.347)	(0.387)
Age 13–16	68.7%	65.6%	68.3%
	(0.464)	(0.475)	(0.465)
Age 17–18	12.2%	20.5%	13.3%
	(0.327)	(0.403)	(0.340)
*Grade*			
Grade 7–8	71.3%	10.9%	62.8%
	(0.452)	(0.311)	(0.483)
Grade 9–11	28.7%	89.1%	37.2%
	(0.452)	(0.311)	(0.483)
Anti-Smoking Media Message	57.7%	58.5%	57.8%
	(0.494)	(0.493)	(0.494)
Knows Tobacco Harms	52.6%	65.7%	54.4%
	(0.499)	(0.475)	(0.498)
Class Discussion	52.1%	32.0%	49.3%
	(0.500)	(0.467)	(0.500)
Ever Offered Free Cigarette	14.6%	16.5%	14.8%
	(0.353)	(0.371)	(0.356)
Exposure to Cigarette Adverts	73.0%	71.0%	72.7%
	(0.444)	(0.454)	(0.446)
*Survey Year*			
Survey Year 2000	10.3%	100.0%	23.0%
	(0.303)	(0.000)	(0.421)
Survey Year 2006	89.7%		77.0%
	(0.303)		(0.421)
*Weekly Pocket Money*			
Lowest Pocket Money	49.1%	66.7%	51.6%
	(0.500)	(0.471)	(0.500)
Low Pocket Money	19.3%	14.9%	18.7%
	(0.395)	(0.356)	(0.390)
High Pocket Money	12.7%	13.6%	12.8%
	(0.333)	(0.343)	(0.334)
Highest Pocket Money	18.9%	048.2%	16.9%
	(0.392)	(0.214)	(0.375)

**Table 2:** Logit models of a 30-day cigarette smoking among youth (aged 11–18) in Ghana and Nigeria (standard errors in parentheses).

Regressors	Estimates for Ghana Only (*N* = 9432)	Estimates for Nigeria Only (*N* = 1555)	Estimates for Both Countries (*N* = 10,987)
	Dataset (1)	Dataset (2)	Dataset (1) +_Dataset (2)
Inflation-Adjusted Price (in LCU)	−0.0806 ***	−0.000871 ***	
	(0.031)	(0.000)	
PPP-Adjusted Price (in I$)			−0.0215 ***
			(0.007)
Gender (Male = 1)	0.0225 ***	0.0239 **	0.0228 ***
	(0.007)	(0.012)	(0.006)
*Age*			
Age 13–16	0.000646	−0.0236	−0.000304
	(0.010)	(0.039)	(0.009)
Age 17–18	0.0101	0.0120	0.0108
	(0.010)	(0.031)	(0.009)
*Grade*			
Grade 9–11	0.0174 *	0.0420	0.0173 *
	(0.009)	(0.032)	(0.009)
*Weekly Pocket Money*			
Low Pocket Money	0.0475 ***	0.0866 ***	0.0506 ***
	(0.006)	(0.023)	(0.006)
High Pocket Money	0.0617 ***	0.119 ***	0.0667 ***
	(0.018)	(0.022)	(0.017)
Highest Pocket Money	0.0406 ***	0.165 ***	0.0474 ***
	(0.010)	(0.018)	(0.010)
At least One Parent Smokes	0.0874 ***	0.0742 ***	0.0871 ***
	(0.007)	(0.005)	(0.007)
Knows Tobacco Harms	−0.0229 ***	−0.0504 ***	−0.0257 ***
	(0.007)	(0.004)	(0.007)
Anti-Smoking Media Message	−0.0121 *	−0.00527	−0.0122 *
	(0.007)	(0.016)	(0.006)
Class Discussion	−0.0291 ***	0.00131	−0.0271 ***
	(0.005)	(0.011)	(0.005)
Exposure to Cigarette Adverts	0.0435 ***	−0.00421	0.0398 ***
	(0.011)	(0.012)	(0.010)
Ever Offered Free Cigarette	0.0389 ***	0.0701 ***	0.0422 ***
	(0.005)	(0.004)	(0.005)
Survey Wave			
Survey Year 2006	−0.00179		−0.0164 **
	(0.013)		(0.008)
Dummy for Nigeria			−0.00384
			(0.006)
Panel B: Elasticity			
Inflation-Adjusted Price (in LCU)	−1.126 **	−0.440 ***	
	(0.463)	(0.134)	
PPP-Adjusted Price (in I$)			−0.900 ***
			(0.337)

Robust clustered-standard errors in parentheses * *p* < 0.1, ** *p* < 0.05, *** *p* < 0.01.

**Table 3:** Characteristics of youth (aged 8–18) in Ghana and Nigeria for a survival model of tobacco use initiation (standard deviations in parenthesis).

Variables	Summary for Ghana Only (*N* = 107,880)	Summary for Nigeria Only (*N* = 15,669)	Summary for Both Countries (*N* = 123,549)
	Dataset (1)	Dataset (2)	Dataset (1) + Dataset (2)
Initiated at one point (or Failure)	4.96%	3.32%	4.75%
	(0.217)	(0.179)	(0.213)
CPI-Adjusted Price (in LCU)	1.756	277.4	
	(0.115)	(40.85)	
PPP-Adjusted Price (in I$)			4.205
			(1.392)
Gender (Male = 1)	53.1%	49.3%	52.6%
	(0.499)	(0.500)	(0.499)
Age (in years)	11.24	11.94	11.33
	(2.492)	(2.568)	(2.513)
Grade (in years of education)	4.458	6.344	4.697
	(2.444)	(2.442)	(2.523)
At least One Parent Smokes	10.3%	12.0%	10.5%
	(0.304)	(0.324)	(0.307)
Anti-Smoking Media Message	56.3%	54.85%	56.1%
	(0.496)	(0.498)	(0.496)
Knows Tobacco Harms	53.9%	71.3%	56.1%
	(0.498)	(0.452)	(0.496)
Class Discussion	55.3%	43.6%	53.8%
	(0.497)	(0.496)	(0.499)
Exposure to Cigarette Adverts	75.0%	65.3%	73.8%
	(0.433)	(0.476)	(0.440)
Ever Offered Free Cigarette	11.8%	12.1%	11.9%
	(0.323)	(0.326)	(0.324)
*Survey Wave*			
Survey Year 2000	6.58%	53.5%	12.5%
	(0.248)	(0.499)	(0.331)
Survey Year 2006	50.7%		44.3%
	(0.500)		(0.497)
Survey Year 2008		46.5%	5.89%
		(0.499)	(0.236)
Survey Year 2009	42.7%		37.3%
	(0.495)		(0.484)
Inflation-Adjusted GDP/capita (in LCU)	770.7	106312.0	
	(264.1)	(29401.9)	
GDP/capita in PPP (in $IS)			2567.9
			(431.8)
Dummy for Nigeria			12.7%
			(0.333)
Individuals	18,231	2678	20,909

**Table 4:** Complementary log-log (cloglog) survival models of tobacco use initiation among youth (ages 8–18) in Ghana and Nigeria (standard errors in parentheses).

Regressors	Estimates for Ghana Only (*N* = 107,880)	Estimates for Nigeria Only (*N* = 15,669)	Estimates for Both Countries (*N* = 123,549)
	Dataset (1)	Dataset (2)	Dataset (1) + Dataset (2)
Panel A: Marginal Effects			
CPI-Adjusted Price (in LCU)	−0.0911 ***	−0.000153 ***	
	(0.016)	(0.000)	
PPP-Adjusted Price (in I$)			−0.0361 ***
			(0.007)
Gender (Male = 1)	0.00291	0.0134 ***	0.00337
	(0.003)	(0.005)	(0.003)
Age (in years)	−0.0119 ***	−0.00527	−0.0112 ***
	(0.004)	(0.004)	(0.003)
Grade (in years of education)	0.00518 ***	0.00371 **	0.00419 ***
	(0.001)	(0.002)	(0.002)
At least One Parent Smokes	0.0389 ***	0.0239 ***	0.0383 ***
	(0.002)	(0.003)	(0.002)
Anti-Smoking Media Message	−0.00299	−0.00469	−0.00310 *
	(0.002)	(0.003)	(0.002)
Knows Tobacco Harms	−0.0151 ***	−0.0152 ***	−0.0149 ***
	(0.003)	(0.003)	(0.003)
Class Discussion	−0.0144 ***	0.00190	−0.0138 ***
	(0.002)	(0.002)	(0.002)
Exposure to Cigarette Adverts	0.0278 ***	0.0115 ***	0.0271 ***
	(0.003)	(0.003)	(0.003)
Ever Offered Free Cigarette	0.0393 ***	0.0302 ***	0.0391 ***
	(0.003)	(0.004)	(0.003)
*Survey Wave*			
Survey Year 2006	0.0147 ***		0.00585
	(0.004)		(0.006)
Survey Year 2008		−0.0176	−0.0971 ***
		(0.013)	(0.036)
Survey Year 2009	0.0128 ***		0.00189
	(0.004)		(0.008)
Inflation-Adjusted GDP/capita in LCU	0.0000254 ***	0.000000239	
	(0.000)	(0.000)	
Log (time)	0.00692	0.0138	0.00571
	(0.012)	(0.015)	(0.012)
GDP/capita in PPP (in I$)			0.0000382 **
			(0.000)
Dummy for Nigeria			0.127 ***
			(0.025)
Panel B: Elasticities			
CPI-Adjusted Price (in LCU)	−3.662 ***	−1.040 **	
	(0.618)	(0.433)	
PPP-Adjusted Price (in I$)			−2.770 ***
			(0.477)
Individuals	18,231	2678	20,909

Robust clustered-standard errors in parentheses * *p* < 0.1, ** *p* < 0.05, *** *p* < 0.01.

**Table 5:** Comparison of results from the complementary log-log (cloglog) survival models to that of split population survival models of tobacco use initiation among youth (ages 8–18) in Ghana & Nigeria (standard errors in parentheses).

Regressors	Estimates for Ghana Only (*N* = 107,880)		Estimates for Nigeria Only (*N* = 15,669)		Estimates for Both Countries (*N* = 123,549)	
	Cloglog	Split Pop.	Cloglog	Split Pop.	Cloglog	Split Pop.
CPI—Adjusted Price (in LCU)	−2.113 ***	−2.090 ***	−0.00472 ***	−0.00469 ***		
	(0.3063)	(0.1384)	(0.0016)	(0.0018)		
PPP—Adjusted Price (in $IS)					−0.642 ***	−0.607 ***
					(0.0436)	(0.0509)
Gender (Male = 1)	0.0166	−0.00728	0.423 ***	0.438 ***	0.0522 **	−0.0295
	(0.0537)	(0.0311)	(0.1467)	(0.0949)	(0.0263)	(0.0317)
Age	−0.152 ***	−0.199 ***	−0.230 ***	−0.233 ***	−0.140 ***	−0.108 ***
	(0.0551)	(0.0222)	(0.0857)	(0.0581)	(0.0188)	(0.0209)
Grade	0.139 ***	0.177 ***	0.121 **	0.127 ***	0.135 ***	0.0813 ***
	(0.0241)	(0.0110)	(0.0511)	(0.0369)	(0.0110)	(0.0141)
At least One Parent Smokes	0.918 ***	1.036 ***	0.741 ***	0.776 ***	0.896 ***	0.989 ***
	(0.0452)	(0.0387)	(0.1243)	(0.1162)	(0.0307)	(0.0390)
Anti-Smoking Media Message	−0.120 ***	−0.110 ***	−0.105	−0.110	−0.121 ***	−0.135 ***
	(0.0434)	(0.0313)	(0.1097)	(0.0942)	(0.0266)	(0.0320)
Knows Tobacco Harms	−0.444 ***	−0.523 ***	−0.494 ***	−0.503 ***	−0.445 ***	−0.485 ***
	(0.0831)	(0.0327)	(0.0881)	(0.0959)	(0.0277)	(0.0329)
Class Discussion	−0.335 ***	−0.375 ***	0.0581	0.0568	−0.305 ***	−0.345 ***
	(0.0441)	(0.0314)	(0.0663)	(0.0951)	(0.0267)	(0.0321)
Exposure to Cigarette Adverts	0.668 ***	0.718 ***	0.295 ***	0.299 ***	0.623 ***	0.652 ***
	(0.0447)	(0.0458)	(0.1116)	(0.1114)	(0.0395)	(0.0456)
Ever Offered Free Cigarette	0.886 ***	1.006 ***	0.922 ***	0.955 ***	0.897 ***	0.984 ***
	(0.0467)	(0.0369)	(0.0857)	(0.1129)	(0.0295)	(0.0374)
Survey Wave						
Survey Year 2006	0.520 ***	0.594 ***			0.493 ***	1.919 ***
	(0.0960)	(0.0721)			(0.0731)	(0.1415)
Survey Year 2008			−0.214	−0.171	−0.467**	−0.782***
			(0.3816)	(0.2536)	(0.1897)	(0.2756)
Survey Year 2009	0.499 ***	0.533 ***			0.483 ***	2.014 ***
	(0.0836)	(0.0789)			(0.0888)	(0.1551)
Inflation-Adjusted GDP/capita in LCU	0.000104	0.000175 *	0.00000201	0.00000194		
	(0.0002)	(0.0001)	(0.0000)	(0.0000)		
Log (time)	−0.378 *	−0.221 ***	0.597 **	0.629 ***	−0.372 ***	−0.201 ***
	(0.2073)	(0.0643)	(0.2869)	(0.2388)	(0.0580)	(0.0634)
GDP/capita in PPP (I$)					−0.000109	0.000320 **
					(0.0001)	(0.0002)
Dummy for Nigeria					2.737 ***	3.320 ***
					(0.2280)	(0.2879)
Constant	1.164 *	1.553 ***	−1.550	−1.474 *	0.113	−2.498 ***
	(0.5942)	(0.2733)	(1.0409)	(0.8440)	(0.3400)	(0.4147)
cure_p						
Constant		−1.128 ***		−1.990		−1.083 ***
		(0.0973)		(1.3502)		(0.1140)
Individuals	18,231	18,231	2678	2678	20,909	20,909

Robust clustered-standard errors in parentheses * *p* < 0.1, ** *p* < 0.05, *** *p* < 0.01

**Table 6:** Effect of PPP-adjusted cigarette price (in I$) on cigarette smoking and tobacco use initiation using a country-specific dataset.

Regressors	Cigarette Smoking		Tobacco Use Initiation	
	Estimates for Ghana Only (*N* = 9432)	Estimates for Nigeria Only (*N* = 1555)	Estimates for Ghana Only (*N* = 107,880)	Estimates for Nigeria Only (*N* = 15,669)
PPP-Adjusted Price (I$)	−0.0224 ***	−0.0149 ***	−0.0468 ***	−0.00704 ***
	(0.009)	(0.005)	(0.008)	(0.002)
Gender (Male = 1)	0.0225 ***	0.0239 **	0.00302	0.0134 ***
	(0.007)	(0.012)	(0.003)	(0.005)
*Age*				
Age 13–16	0.000635	−0.0236		
	(0.010)	(0.039)		
Age 17–18	0.0100	0.0120		
	(0.010)	(0.031)		
*Grade*				
Grade 9–11	0.0174 *	0.0420		
	(0.009)	(0.032)		
*Weekly Pocket Money*				
Low Pocket Money	0.0475 ***	0.0866 ***		
	(0.006)	(0.023)		
High Pocket Money	0.0617 ***	0.119 ***		
	(0.018)	(0.022)		
Highest Pocket Money	0.0406 ***	0.165 ***		
	(0.010)	(0.018)		
At least One Parent Smokes	0.0874 ***	0.0742 ***	0.0390 ***	0.0238 ***
	(0.007)	(0.005)	(0.002)	(0.003)
Anti-Smoking Media Message	−0.0121*	−0.00527	−0.00310	−0.00461
	(0.007)	(0.016)	(0.002)	(0.003)
Knows Tobacco Harms	−0.0229 ***	−0.0504 ***	−0.0147 ***	−0.0155 ***
	(0.007)	(0.004)	(0.003)	(0.003)
Class Discussion	−0.0291 ***	0.00131	−0.0142 ***	0.00188
	(0.005)	(0.011)	(0.002)	(0.002)
Exposure to Cigarette Adverts	0.0435 ***	−0.00421	0.0277 ***	0.0117 ***
	(0.011)	(0.012)	(0.003)	(0.003)
Ever Offered Free Cigarette	0.0390 ***	0.0701 ***	0.0391 ***	0.0302 ***
	(0.005)	(0.004)	(0.003)	(0.004)
*Survey Wave*				
Survey Year 2006	−0.0161 **		−0.000163	
	(0.008)		(0.006)	
Survey Year 2008				−0.00383
				(0.012)
Survey Year 2009			−0.00900	
			(0.007)	
Age			−0.0117 ***	−0.00546
			(0.004)	(0.004)
Grade			0.00290 *	0.00454 **
			(0.002)	(0.002)
GDP/capita in PPP (I$)			0.0000680 ***	−0.00000122
			(0.000)	(0.000)
Log (time)			0.00624	0.0148
			(0.012)	(0.015)

Robust clustered-standard errors in parentheses * *p* < 0.1, ** *p* < 0.05, *** *p* < 0.01.
